# Guillain-Barre syndrome related to SARS-CoV-2 vaccinations

**DOI:** 10.1016/j.clinsp.2022.100113

**Published:** 2022-09-12

**Authors:** Josef Finsterer, Carla A. Scorza, Fulvio A. Scorza

**Affiliations:** a Neurology & Neurophysiology Center, Vienna, Austria; b Disciplina de Neurociência, Universidade Federal de São Paulo, Escola Paulista de Medicina (UNIFESP/EPM), São Paulo, SP, Brasil

**Keywords:** AIDP, acute, inflammatory demyelinating polyneuropathy, AMAN, acute, motor, axonal neuropathy, AMSAN, acute, motor, and sensory axonal neuropathy, AZV, Astra Zeneca vaccine, CNS, central nervous system, FDA, Food and drug administration, GBS, Guillain Barres syndrome, IVIG, ntravenous Immunoglobulins, MHRA, Medicine and Healthcare products Regulatory Agency, PCB, Pharyngo-Cervico-Brachial, PE, plasma exchange, PNS, pcripheral nervous system, SC2VaG, SARS-CoV-2 vaccination associated GBS

*Dear Editor*,

There are indications that SARS-CoV-2 vaccinations can be complicated by impairment of the Central or Peripheral Nervous System (CNS, PNS).^1^^,^^2^ One of the most frequent PNS complications of SARS-CoV-2 vaccinations is Guillain-Barre syndrome (GBS, SC2VaG).^3^ In a recent review, SC2VaG has been reported in 19 patients collected until the end of June 2021.^4^ Additionally, > 300 SC2VaG patients had been reported by the FDA and EMA.^4^ Between the first of July and the end of September 2021 further SC2VaG patients have been published. This narrative review aimed at summarising previous and recent findings regarding the clinical presentation, therapeutic management, and outcome of patients with SC2VaG collected until the end of September 2021.

A literature search in the databases PubMed and Google Scholar over the period January 2020 until the end of September 2021 using the search terms “neuropathy”, “Guillain Barre syndrome”, “polyradiculitis”, “AIDP”, “AMAN”, “AMSAN”, “Miller-Fisher syndrome”, “polyneuritis cranialis”, “Pharyngo-Cervico-Brachial (PCB)”, and “Bickerstaff encephalitis”, in combination with “SARS-CoV-2”, “COVID-19”, “vaccination”, “immunization”, and “coronavirus” was conducted. Additionally, reference lists were checked for further articles meeting the search criteria. Included were original studies detailing individual patients’ data (age, sex, latency between vaccination and SC2VaG, GBS subtype, treatment, and outcome) and large cohort studies mentioning the number of SC2VaG patients without detailing individual patients’ data (pooled data). Excluded from the analysis were reviews, abstracts, proceedings, and editorials.

Altogether 23 articles detailing individual data from 52 SC2VaG patients were retrieved. Additionally, 5 studies reporting pooled data of 337 SC2VaG cases without individual details were included (Table 1). Pooled data were available from 96 SC2VaG cases following vaccinations with the Johnson & Johnson vaccine (JJ),^5^ from 7 patients after the first Pfizer jab,^6^ from 1 patient after vaccination with the JJ vaccine,^7^ from 227 patients after vaccination with the AZV,^8^ and from 6 patients reported in the Medicine and Healthcare products Regulatory Agency (MHRA) database (Table 1). Evaluating the 52 patients from whom individual data were available, age, reported in 50 cases, ranged between 7‒90y (Table 1). Gender was reported in 50 cases among which 29 were male and 21 female (Table 1). The number of jabs was reported in 50 patients. SC2VaG developed after the first shot in 46 patients and after the second shot in four patients (Table 1). The latency between vaccination and onset of SC2VaG ranged between 3h and 39d (Table 1). The type of vaccine was reported in 50 patients. AZV was given to 39 patients, Pfizer to 9 patients, and JJ to 2 patients. Therapy of SC2VaG was reported in 34 patients (Table 1). Intravenous Immunoglobulins (IVIG) were given to 28 patients, steroids to four patients, and plasma exchange was applied to 3 patients. Two patients only received gabapentin (Table 1). One patient did not receive any treatment at all. Eight patients required mechanical ventilation. None of the patients died but the complete recovery could be achieved in only four patients. Partial recovery was reported in 23 patients (Table 1). In the remainder of the observation, the outcome was not mentioned (Table 1).

**Table 1:** Patients with GBS following a SARS-CoV-2 vaccination published as per the end of September 2021.

Age		Sex	1/2 dose	Vaccine	LVG	Treatment	MV	Outcome
**Individual data**								
90		m	Second	Pfizer	3d	IVIG	no	pr
51		f	First	AZV	10d	IVIG	no	cr
62		m	First	AZV	15d	IVIG	no	pr
41		m	Nr	JJ	10d	IVIG	no	pr
75		f	First	AZV	< 28d	nr	nr	nr
77		f	First	AZV	< 28d	nr	nr	nr
57		f	First	AZV	< 28d	nr	nr	nr
57		m	First	AZV	< 28d	nr	nr	nr
52		f	First	AZV	< 28d	nr	nr	nr
54		m	First	AZV	< 28d	nr	nr	nr
80		f	First	AZV	< 28d	nr	nr	nr
72		m	First	AZV	< 28d	nr	nr	nr
59		m	First	AZV	< 28d	nr	nr	nr
69		m	First	AZV	< 28d	nr	nr	nr
72		f	First	AZV	< 28d	nr	nr	nr
66		m	First	AZV	< 28d	nr	nr	nr
63		m	First	AZV	< 28d	nr	nr	nr
70		m	First	AZV	< 28d	nr	nr	nr
38		m	Nr	nr	14d	IVIG	no	pr
47		m	First	AZV	17d	IVIG	no	pr
Nr		nr	First	Pfizer	Nr	nr	no	nr
48		m	First	AZV	10d	St, IVIG	no	pr
51		m	First	AZV	14d	IVIG, PE	MV	pr
65		f	First	AZV	7d	IVIG	no	pr
72		m	First	AZV	21d	IVIG	no	pr
66		m	First	AZV	21d	IVIG	no	pr
Nr		nr	Second	Pfizer	Nr	nr	no	nr
65		m	First	Pfizer	2d	IVIG	no	cr
58		m	First	AZV	3d	GBT	no	pr
37		f	First	AZV	4d	GBT	no	pr
76		m	Second	nr	14d	IVIG	no	cr
73		m	Second	Pfizer	20d	IVIG	no	pr
62		f	First	AZV	8d	IVIG	MV	pr
54		m	First	AZV	12d	St	no	nr
20		m	First	AZV	21d	St	no	nr
57		m	First	AZV	11d	IVIG	no	nr
55		m	First	AZV	22d	none	no	nr
32		m	First	AZV	8d	IVIG^a^	no	pr
69		f	First	AZV	39d	IVIG	no	cr
86		f	First	Pfizer	1d	IVIG	no	pr
82		f	First	Pfizer	14d	IVIG	no	pr
43		f	First	AZV	10d	IVIG	MV	nr
67		f	First	AZV	14d	IVIG	MV	nr
53		f	First	AZV	12d	nr	MV	nr
68		f	First	AZV	14d	nr	MV	nr
70		m	First	AZV	11d	IVIG	MV	pr
69		f	First	AZV	12d	IVIG, PE	no	pr
69		f	First	AZV	13d	IVIG	MV	pr
52		f	First	Pfizer	3h	St, PGB	no	pr
77		m	First	Pfizer	3d	IVIG, PE	no	nr
7		m	First	AZV	14d	IVIG	no	pr
60		f	First	JJ	17d	IVIG	no	pr
**Pooled data**								
Nr	61m		nr	JJ	< 42d	nr	nr	nr (n = 96)
Nr	nr		First	Pfizer	< 30d	nr	nr	nr (n = 7)
Nr	nr		First	JJ	Nr	nr	nr	nr (n = 1)
Nr	nr		nr	nr	Nr	nr	nr	nr (n = 6)
Nr	nr		nr	AZV	Nr	nr	nr	nr (n = 227)

AZV, Astra Zeneca Vaccine, cr, complete recovery, f, female, LVG, Latency between Vaccination date and onset of GBS, m, male, GBT, Gabapentin, JJ, Johnson & Johnson vaccine, MV, Mechanical Ventilation, nr, not reported, PGB, Pregabalin, pr, partial recovery, PE, Plasma Exchange, St, Steroids.

a The patient received two cycles, plasmapheresis, and is now undergoing immune adsorption, (%) the patient had CIDP.

This narrative literature review of the databases PubMed and Google Scholar shows that the number of SC2VaG patients is much higher than anticipated. As of the end of September 2021 at least 389 patients with SC2VaG have been reported. In the vast majority of the cases, SC2VaG occurred after the first jab. In most cases, SC2VaG occurred within 14 days after the vaccination. In the vast majority SC2VaG developed after immunization with AZV, followed by JJ, and Pfizer. Though none of the SC2VaG patients died from the vaccination, SC2VaG included the respiratory muscles in eight patients who required mechanical ventilation.

Though a causal relationship between the vaccinations and GBS cannot be established with this study, the high number of GBS cases within nine months since introduction of the vaccines cannot be neglected and suggests that rarely vaccinations can be complicated by GBS. Clinical presentation, treatment, and outcome of SC2VaG do not seem to be at variance from GBS triggered by other causes. Whether previous affection of peripheral nerves due to causes other than SARS-CoV-2 predispose for the development of SC2VaG remains speculative but currently, there are no indications that patients with pre-existing polyneuropathy or small fiber neuropathy are at an increased risk for SC2VaG. The only possible risk factor for acquiring SC2VaG seems to be a history of a previous GBS.^9^ SC2VaG is a serious complication of SARS-CoV-2 vaccines^10^ and requires prompt diagnostic confirmation and initiation of adequate treatment to prevent rapid progression with involvement of the respiratory muscles and poor outcome. Though SARS-CoV-2 vaccinations may trigger SC2VaG, vaccinations seem to have reduced the prevalence of SARS-CoV-2 infection-related GBS.^11^

In conclusion, this review provides evidence that there is a causal relation between SARS-CoV-2 vaccinations and GBS. Those involved in the management of SARS-CoV-2 vaccinations and their complications should be aware of SC2VaG as a severe complication of the vaccinations. Before patients are vaccinated, they should be screened for potential risk factors and should be informed about this potential complication, and basic research is needed to uncover the pathophysiological mechanisms underlying SC2VaG.

## Authors' contributions

JF: design, literature search, discussion, first draft, critical comments; FS and CS: literature search, discussion, critical comments, final approval.

## Ethical approval and consent to participate

Not applicable.

## Consent for publication

Not applicable.

## Conflicts of interest

The authors declare no conflicts of interest.
